# Patient perspectives on exercise among adults with postural orthostatic tachycardia syndrome: a mixed methods study

**DOI:** 10.1007/s10286-025-01166-0

**Published:** 2025-10-30

**Authors:** Elizabeth G. Walsh, Gurjeet S. Birdee, Kemberlee Bonnet, David G. Schlundt, Chandler Broadbent, Erin C. Kelly, Kayleigh Rogalski, Kristin R. Archer, Alfredo Gamboa

**Affiliations:** 1 Department of Physical Medicine & Rehabilitation, The Osher Center for Integrative Health at Vanderbilt, Vanderbilt University Medical Center, 3401 West End Ave, Ste 380, Nashville, TN 37203 USA; 2 Department of Psychology, Vanderbilt University, Nashville, TN USA; 3 Department of Psychiatry and Behavioral Sciences, Vanderbilt University Medical Center, Nashville, TN USA; 4 Division of Pain, Department of Anesthesiology, Vanderbilt University Medical Center, Nashville, TN USA; 5 Department of Orthopaedic Surgery, Vanderbilt Center for Musculoskeletal Research, Vanderbilt University Medical Center, Nashville, TN USA; 6 Vanderbilt Autonomic Dysfunction Center, Vanderbilt University Medical Center, Nashville, TN USA; 7 Division of Clinical Pharmacology, Vanderbilt University Medical Center, Nashville, TN USA; 8 Department of Medicine, Vanderbilt University Medical Center, Nashville, TN USA

**Keywords:** POTS, Postural orthostatic tachycardia syndrome, Exercise, Qualitative

## Abstract

**Purpose:**

Despite the central role of exercise in treating postural orthostatic tachycardia syndrome (POTS) there have been no studies on the subjective experience of exercise interventions and/or recommendations among this patient population. The purpose of this mixed-methods study was to provide greater understanding of the perceived barriers, preferences, perceptions of exercise, and experiences implementing exercise recommendations for adults with POTS in order to optimize treatment recommendations and intervention design.

**Methods:**

This study consisted of a series of focus groups (*n* = 29) and an online survey of adults with POTS (*n* = 255) focusing on exercise engagement, beliefs, barriers, and facilitators. Qualitative data were analyzed using an iterative inductive-deductive approach, informed by social cognitive theory, which resulted in a conceptual framework and a series of themes.

**Results:**

Survey results showed that participants reported a wide range of exercise frequency prior to the onset of POTS symptoms, and overall lower exercise engagement post-POTS. In both survey results and qualitative findings, participants reported believing that exercise is important in managing POTS, but identified barriers to exercise training, including most saliently, their symptom burden. Participants also identified important needs and facilitating factors that could support them in engaging in regular exercise to help manage their condition.

**Conclusion:**

These findings shed light on the patient experience of exercise in POTS, which can inform both the tailoring of exercise recommendations and the design of interventions to support exercise engagement specific to the POTS population.

## Introduction

Postural orthostatic tachycardia syndrome (POTS) is the most common autonomic disorder, affecting up to 1% of the U.S. population, with a strong preponderance among white women of childbearing age [1]. There is broad consensus that POTS is a “final common pathway” for heterogenous pathophysiological processes, including partial sympathetic neuropathy, hypovolemia, mast cell activation, autoimmunity, and severe cardiovascular deconditioning, with possible distinct phenotypes based on underlying pathology [2, 3]. However, these processes and how they interact are not completely understood and treatments largely target symptoms rather than underlying pathophysiological processes.

Exercise is considered a first-line treatment for POTS and is recommended regardless of presumed etiology. While the first studies of exercise in POTS framed it as countering deconditioning, there has since been significant debate as to whether deconditioning is a primary cause of POTS or is more likely to occur secondary to the disorder [3]. Furthermore, research findings examining the prevalence of deconditioning in individuals diagnosed with POTS have varied greatly [4–7], with those investigating recumbent (vs. upright) exercise not finding evidence of deconditioning [6, 8]. Studies of the pathophysiology of exercise intolerance in individuals with POTS have suggested other mechanisms, including cerebral hypoperfusion, decreased venous return, breathing dysfunction, and muscle abnormalities [9]. While there is not consensus about the role deconditioning plays in the etiology and pathophysiology of POTS, exercise is considered to be vital for its capacity to counteract orthostatic intolerance by increasing blood volume and left ventricular mass, and improving vascular compression and endothelial function [5, 10].

Only a handful of studies have directly examined the effectiveness of exercise for POTS, finding consistent and significant improvements in orthostatic hemodynamics among completers of exercise programs [11–14]. Unfortunately, these studies reported high non-completion rates (i.e., 44–59%), highlighting the importance of patient adherence to a behaviorally intensive treatment such as exercise and raising the possibility that study results may be biased towards those with better exercise tolerance [15]. Indeed, adherence is a major concern with physical activity engagement, both in the general population and among those with chronic health conditions. Only a quarter of the general population of U.S. adults report the amount of regular exercise recommended by the U.S. Centers of Disease Control and Prevention (CDC), despite its well-identified benefits for general health [16]. In addition to the barriers faced by the general population, individuals with POTS are likely to experience condition-specific barriers such as exercise intolerance, heat intolerance, and fatigue. A retrospective case review of females with POTS completing a personalized cardiac rehabilitation program reported ongoing symptoms of postural intolerance as a major barrier to adherence [17].

The purpose of this mixed-methods study is to provide greater understanding of the perceived barriers, preferences, perceptions of exercise, and experiences implementing exercise recommendations for adults with POTS. Despite the central role exercise is thought to play in treating POTS, to our knowledge there have been no phenomenological explorations of the patient experience of exercise interventions and/or recommendations among this patient population. Findings from an online survey and focus groups will provide valuable insights to inform the optimization of treatment approaches.

## Methods

### Study design and participants

Participants were recruited through the Vanderbilt Medical Center (VUMC) and online for a focus group and web-based survey. VUMC is a major academic medical center that serves as a national referral for POTS patients through the Autonomic Dysfunction Center and the Osher Center for Integrative Health. Flyers were posted at the Osher Center, and online recruitment occurred through ResearchMatch, social media via patient organizations (Dysautonomia International and Standing Up to POTS), and a VUMC research listserv. ResearchMatch is a national health volunteer registry that was created by several academic institutions, originally hosted by VUMC and supported by the U.S. National Institutes of Health as part of the Clinical Translational Science Award (CTSA) program [18]. Inclusion criteria were English-speaking adults (age > 18 years) with a self-reported diagnosis of POTS. Exclusion criteria were factors that could interfere with participation and included severe cognitive impairment, moderate to severe or unresolved traumatic brain injury, and severe mental illness (i.e., schizophrenia, psychotic disorder).

### Study procedures

All study procedures and materials were reviewed and approved by the Institutional Review Board at VUMC (IRB # 211754). Survey data were collected and managed using REDCap electronic data capture tools hosted at VUMC [19, 20]. Survey responses were collected between September and December 2022, and then again from February through August 2024. Focus groups were completed October through December 2022. Study advertisements directed interested individuals first to a REDCap survey to complete an e-Consent and eligibility forms, following which they were directed automatically to an online survey and then to a focus group scheduling form (only during the period of data collection during which focus groups occurred).

#### Online survey

The online survey consisted of a brief demographic and background questionnaire, as well as study-specific items assessing the use of lifestyle modifications, experience with and beliefs about exercise as a treatment for POTS, and degree of functional disability. Quantitative data were managed by REDCap and analyzed using SPSS version 27 (SPSS IBM Corp., Armonk, NY, USA).

#### Focus groups

Qualitative data collection, coding, and analysis were managed by the Vanderbilt University Qualitative Research Core (VU-QRC), which is a service core of the Division of Health Services Research in the Department of Medicine and Public Health at Vanderbilt University. The methods were informed by the COREQ (Consolidated criteria for reporting qualitative research), an evidence-based guideline/checklist for qualitative research methods [21]. A focus group guide was developed by the coauthors in collaboration with the VU-QRC and then pilot tested among the study team. It was informed by social cognitive theory (SCT) [22] and included questions about living with POTS, lifestyle management, and exercise. Online focus groups using Zoom were conducted by members of the study team, including at least one member of the VU-QRC. All focus groups were audio-recorded and transcribed using an IRB-approved transcription service (rev.com).

#### Qualitative data coding

A hierarchical coding system was developed and refined using the interview guide and preliminary review of transcripts, consisting of major categories and subcategories, with definitions and rules for each category. Coding was completed by members of the study team trained in coding, and reliability was established among the coders. The coded transcripts were combined and sorted by code. More details on the focus group methodology and coding process as well as the full coding system can be found in Walsh et al. [23].

Additionally, the online survey included an open-ended question, “Is there anything else you would like to share about your experience with doctors recommending exercise?” We received 130 responses, which were coded using the system developed for the focus group and combined with focus group findings for analysis. Transcripts, quotations, and codes were managed using Microsoft Excel 2016 (Microsoft Corp., Redmond, WA, USA) and SPSS version 27 (SPSS IBM Corp.).

#### Qualitative analysis and conceptual model

As there is no extant literature examining exercise beliefs and behavior in POTS, our study design was guided by theory but open enough to allow unexpected themes to emerge. Qualitative analysis followed an iterative inductive/deductive approach [24, 25]. Inductively, we used the coded, sorted quotes to identify themes and relationships between themes. Initial analysis revealed two supra-categories: (1) exercise beliefs and experience and (2) overall experiences of living and navigating healthcare with POTS. The study team agreed to separate these categories for analysis, and findings related to overall experiences were reported separately [23]. Deductively, we were guided by SCT, which also guided the design of the focus group guide and survey questions. SCT is a commonly used theory for understanding exercise behavior and specifies that engagement in a behavior is reciprocally influenced by personal and social factors. Important concepts in SCT include outcome expectations (expectations about the consequences of a specific behavior) and self-efficacy (the belief that one can complete a task or accomplish a goal) [26, 27].

**Fig. 1:**
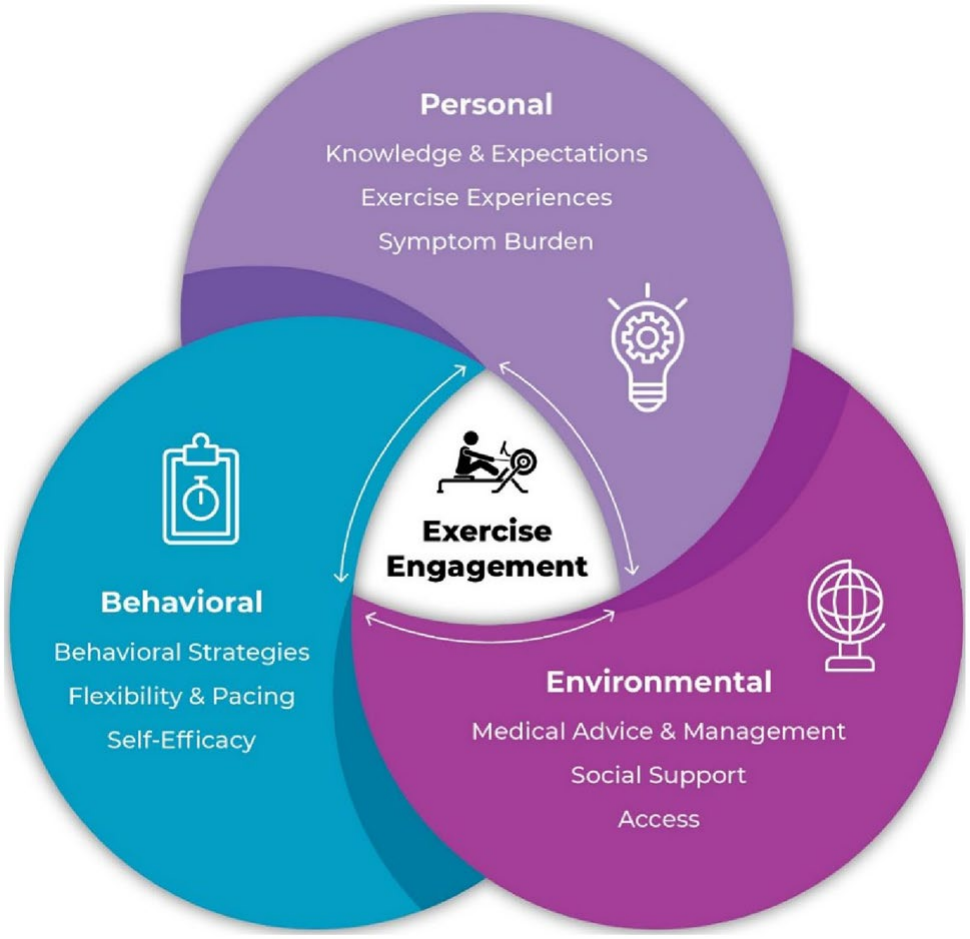
Conceptual model of influences on exercise in postural orthostatic tachycardia syndrome

Using this iterative inductive/deductive approach, we developed a conceptual framework (Fig. 1). This conceptual framework, rooted in SCT, illustrates the complex interplay of factors influencing exercise behavior among individuals with POTS. This model was developed during the analysis phase and then guided the organization and presentation of findings. The model includes three primary domains: (1) personal factors, which are unique to each individual, including knowledge and understanding of the role of exercise in managing POTS and symptom burden; (2) environmental factors include access to exercise facilities and equipment, social support, and medical management and advice; and (3) behavioral factors, which represent elements of behavioral self-regulation, or factors that influence to what degree someone engages in an intended behavior, including self-efficacy for the task and behavioral strategies. The bidirectional arrows in the model represent the principle of reciprocal determinism, highlighting the dynamic relationships among these domains and their collective influence on exercise behavior.

## Results

### Participants

**Fig. 2:**
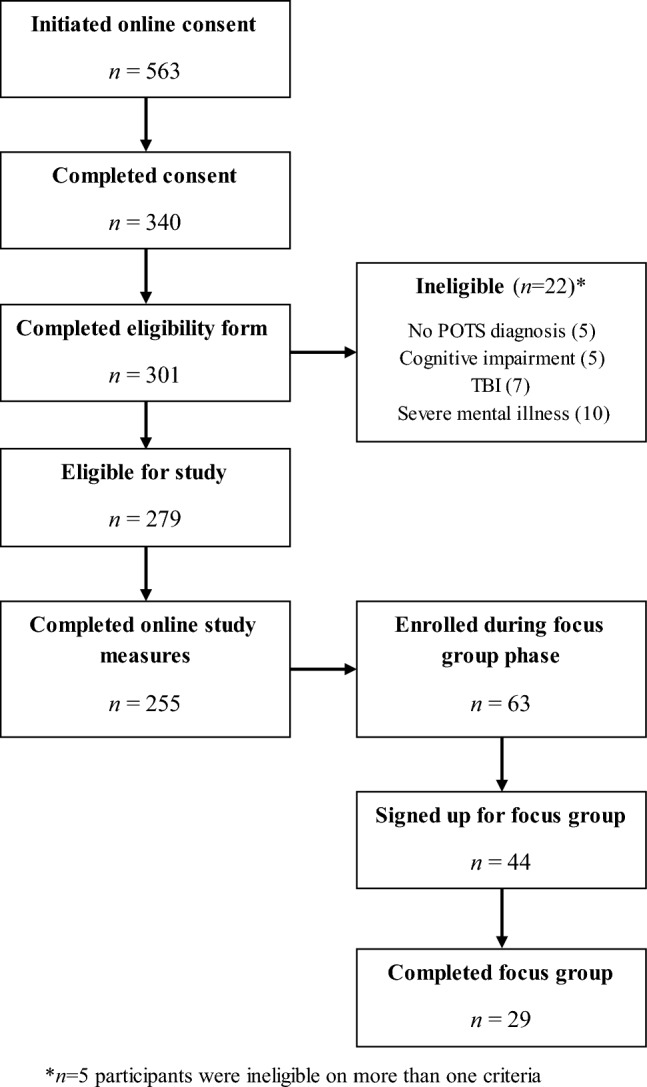
Study flow diagram.* POTS * Postural orthostatic tachycardia syndrome,* TBI* traumatic brain injury

A total of 255 individuals completed online survey measures. Data were included in analyses on all participants who completed any of the study-specific questionnaire (see Fig. 2 for a study flow diagram).

The survey sample was overwhelmingly female (94.9%), majority white (84.3%), with an age range of 18–78 (mean 35.4, standard deviation [SD] 11.53) years. Participants reported an average time to diagnosis of > 6 years. Full descriptive statistics are presented in Table 1. A subset of 29 individuals participated in focus groups. There were no statistically significant differences between the focus group sample and the larger survey sample in terms of mean age, number of comorbidities, reported exercise engagement, or self-reported functional disability.

**Table 1:** Demographic descriptive statistics (*n* = 255)

Variable	Metric
Age in years, mean (SD)	35.36 (11.53)
*Gender, count (%)*	
Female	241 (94.9)
Male	6 (2.4)
Transgender	6 (2.4)
Other	1 (0.4)
Did not disclose	1 (0.4)
*Racial/ethnic identity, count (%)*	
American Indian/Alaskan Native	2 (0.8)
Asian	7 (2.7)
Black or African American	7 (2.7)
Hispanic or Latino	11 (4.3)
White	215 (84.3)
Multiracial	8 (3.1)
Did not disclose	5 (2.0)
*Employment status, count (%)*	
Work full time	101 (39.5)
Work part time (20–40 h/wk)	28 (11.0)
Work part time (< 20 h/wk)	23 (9.0)
Disabled/On disability	33 (12.9)
In school full time	22 (8.6)
In school part time	4 (1.6)
Unemployed/between jobs	43 (16.9)
Did not disclose	1 (0.4)
*Diagnosis*	
Symptom onset age in years, mean (SD)	24.54 (12.42)
Age at diagnosis in years, mean (SD)	31.27 (11.40)
Time to diagnosis (calculated), in years, mean (SD)	6.68 (8.03)
Type of clinician diagnosed, count (%)	
Cardiologist	166 (65.1)
Neurologist	38 (14.9)
Primary care	31 (12.2)
Other	20 (7.8)
*Comorbidities*^*a*^	
Number of diagnosed comorbidities, mean (SD)	1.76 (1.60)
Number of suspected comorbidities, mean (SD)	1.36 (1.29)
Migraine (diagnosed/suspected), count (%)	109 (42.7)/26 (10.2)
ME/CFS (diagnosed/suspected), count (%)	44 (17.3)/76 (29.8)
EDS (diagnosed/suspected), count (%)	56 (22.0)/56 (22.0)
IBS (diagnosed/suspected), count (%)	62 (24.3)/35 (13.7)
MCAS (diagnosed/suspected), count (%)	31 (12.2)/63 (24.7)

*EDS* Ehlers Danlos syndrome, *IBS* irritable bowel syndrome, *MCAS* mast cell activation syndrome, *ME/CFS* myalgic encephalomyelitis,* SD* standard deviation

^a^Survey asked about 10 comorbidities plus “other”; table lists the 5 highest-frequency reported overall (based on combined frequency of diagnosed and suspected)

### Survey results

#### Lifestyle management and exercise engagement

**Fig. 3:**
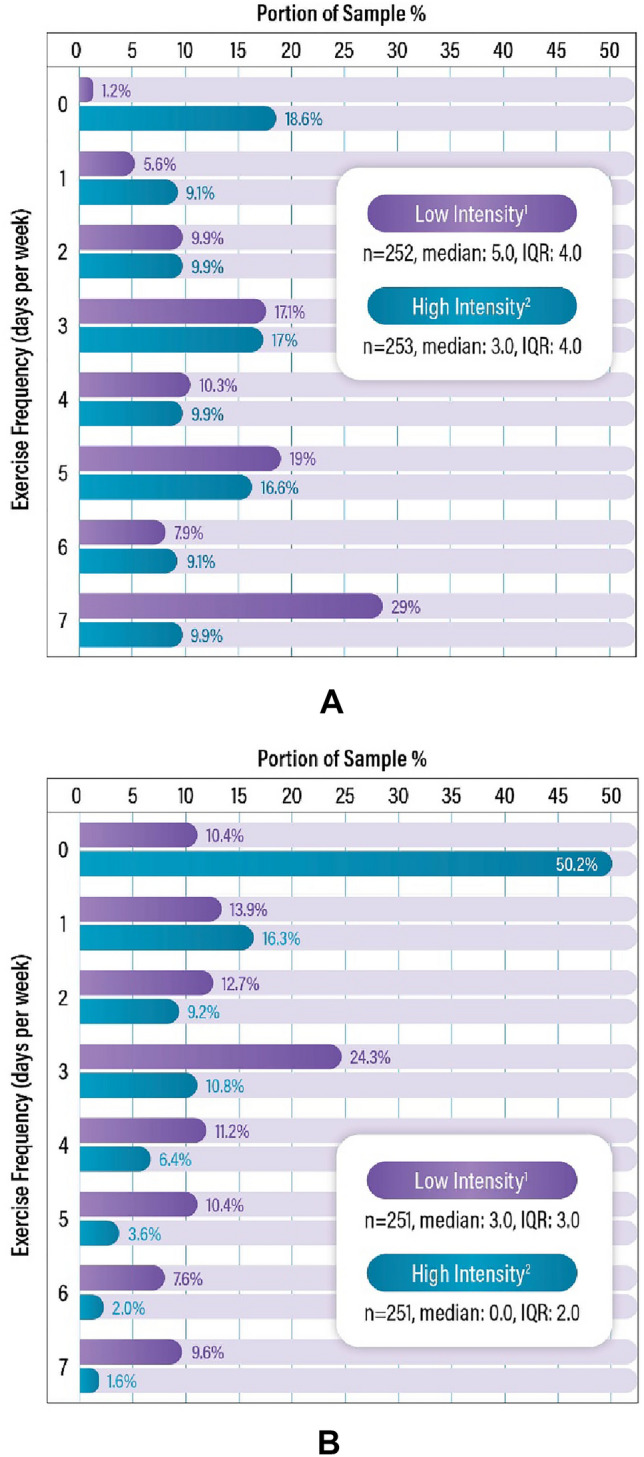
Self-reported frequency of average days per week of exercise. **a** Self-reported frequency (days/week) of exercise prior to the onset of POTS symptoms. **b** Self-reported frequency (days/week) of current exercise (6 months prior to survey completion). ^1^Low-intensity exercise defined as “at least 30 min of low-intensity physical activity (i.e., walking, yoga)”. ^2^High-intensity exercise defined as “at least 30 min of moderate to high-intensity physical activity (i.e., running, cardio machines, high intensity sports)”. * POTS * Postural orthostatic tachycardia syndrome

Distributions of reported exercise prior to the onset of POTS symptoms and currently are shown in Fig. 3. On average, participants reported a higher rate of low-intensity (median 5 days/week, interquartile range [IQR] 4) than high-intensity (median 3 days/week, IQR 4) exercise prior to the onset of POTS symptoms. Average rates of reported exercise were currently lower (“over the past 6 months”) than pre-POTS, with a higher frequency of low-intensity (median  3 days/week, IQR 3) than high-intensity (median 0 days/week, IQR 2) exercise.

Table 2 presents descriptive statistics extracted from the survey. We asked participants about their experience with commonly recommended lifestyle interventions. Most reported having tried all the included interventions, with all except physical counter-maneuvers and eating small meals attempted by > 90% of participants. Avoiding triggers was rated as the most helpful intervention, with > 80% of respondents rating it as at least moderately helpful.

**Table 2:** Survey descriptive statistics

Variable	Metric
*Lifestyle interventions* (1 = not at all helpful, 5 = extremely helpful)^a^	*Median (IQR)*
Increasing water intake	3.0 (2.0)
Increasing salt intake	3.0 (2.0)
Wearing compression garments	2.0 (2.0)
Physical countermaneuvers	2.0 (1.0)
Avoiding triggers	4.0 (2.0)
Eating small meals	3.0 (2.0)
*Functional disability*	*Median (IQR)*
Symptom interference with daily activities (0–10)	7.0 (3.0)
Symptom impairment: social/family activities (0–10)	7.0 (4.0)
Symptom impairment: work activities (0–10)	7.0 (4.75)
Number of days in 6 months symptoms kept you from life activities (0–185)	90.0 (124.0)
*Exercise* (1 = not at all, 5 = extremely)	*Median (IQR)*
How important do you believe exercise is in managing POTS?	4.0 (2.0)
How confident are you that you could engage in regular exercise?	3.0 (1.0)
How confident are you that exercise would help your POTS?	4.0 (1.0)
*Protocols*^b^	*Yes, N (%)*
Are you familiar with the Levine or POTS exercise protocol? (*n* = 255)	172 (67.5)
Has a doctor ever prescribed you the POTS exercise protocol? (*n* = 255)	79 (31.0)
Did you attempt it? (*n* = 79)	68 (86.1)
Did you complete it? (*n* = 76)	28 (36.8)
*Source of knowledge*^c^	*N (%)*
Doctor	63 (24.7)
POTS patient organization	85 (33.3)
Internet search	76 (29.8)
Other	57 (22.4)

*IQR* Interquartile range,* POTS * postural orthostatic tachycardia syndrome

^a^Survey options for lifestyle interventions included option “Haven’t tried it”; medians and IQRs were calculated without these responses to not skew data

^b^Sample sizes vary for these items because the questions about attempting and completing protocol were asked only of those reporting they had been prescribed the protocol. Response options for first two questions included “yes”, “no” and “not sure”

^c^Item asked respondents to “check all that apply”; therefore item total is greater than total sample size (*n* = 255). Percentage is calculated as percentage of sample size to capture proportion of respondents who endorsed each source of knowledge

Overall, participants reported believing that exercise is important in managing POTS, while confidence that regular exercise would improve POTS symptoms was more varied. Confidence about ability to exercise regularly was also varied, with about half of survey respondents indicating they were very or extremely confident they could engage in 30 min of exercise 3 times per week. However, nearly a quarter indicated that they were not very or not at all confident they could exercise regularly.

We asked participants if they were familiar with the “Levine, or POTS exercise protocol” as it is considered the original exercise training protocol for POTS [28]. This protocol outlines a 3-month progression that begins with recumbent cardio exercise combined with resistance training and gradually works up to 45–60 min of upright exercise 5–6 days per week. A majority reported being familiar with this protocol, with the most common source of knowledge being POTS patient networks or internet search, followed by medical providers. However, less than one third of respondents reported having had the protocol prescribed to them. Of these, the majority reported that they had tried it, but only about one third of those attempting it reported completing it.

#### Barriers

Barriers were assessed using an open-ended field asking participants to list their biggest barriers to exercise. Responses from 230 participants were coded using a coding system developed based on review of all responses, and each response was assigned up to six codes. The frequency of barriers listed is presented in Table 3. Symptoms, including fatigue, tachycardia, shortness of breath, and post-exertional malaise, were by far the most frequently listed barriers. Over 90% of responses included at least one symptom or health-related barrier, with fatigue as the most common, reported by > 50% of respondents.

**Table 3:** Barriers as reported in survey (*n* = 230)

Barrier	Frequency (%)^a^
*Health/symptom related*	
Fatigue/exhaustion/lack of energy	117 (50.9)
Comorbid conditions/injuries	63 (27.4)
Dizziness/lightheadedness	57 (24.8)
POTS symptoms (general or misc)	54 (23.5)
Tachycardia/palpitations	41 (17.8)
Exercise triggers flares	31 (13.5)
Heat/temp dysregulation/weather	26 (11.3)
Syncope/fainting	25 (10.9)
Shortness of breath	21 (9.1)
Post-exertional malaise	19 (8.3)
Exercise intolerance	14 (6.1)
Upright posture	5 (2.2)
Any symptom/health related barrier	210 (91.3)
*Situational/psychological barriers*	
Lack of time	35 (15.2)
Other/competing responsibilities	29 (12.6)
Gym access/financial/transportation issues	23 (10.0)
Negative expectations/fear	15 (6.5)
Lack of motivation	11 (4.8)
Don’t like it/it’s uncomfortable	9 (3.9)
Don’t know what to do/need guidance	8 (3.5)
No one to exercise with	6 (2.6)
Not in habit/hard to create habit	5 (2.2)

* POTS * Postural orthostatic tachycardia syndrome

^a^Each item was a free response and therefore respondents could list as many barriers as they chose. Percentage is calculated as the percentage of overall responses (*n* = 230) that included a relevantly coded word/phrase

### Qualitative themes

Themes from the iterative inductive/deductive qualitative analysis are listed and in the following sections, with illustrative quotations included in Table 4. Frequency counts for themes are not included as the iterative inductive/deductive approach is a thematic rather than quantitative approach to analyzing qualitative data [29]. Furthermore, while all themes are derived from coded quotations, themes are not necessarily analogous to codes (e.g., theme of “Diversity in experience, engagement, and preferences” derived from quotations coded “Before POTS” and “Exercise”).

**Table 4:** Themes and illustrative quotations

Quotation	Group: ID/Survey
*Theme 1: Diversity in exercise experience, engagement, and preferences*	
I’ve always been an extremely athletic person… probably like five days out of the week, moderate to intense exercise for 30 to 60 min.	4:1 (High)
I’ve never felt better after exercise since I was a teenager… exercise always made me feel worse. I kept trying, did it for being friends with people and for other reasons. But it was never because the exercise ever made me feel better.	1:3 (Low)
Bad advice would be walking for me, absolutely terrible. Worst thing I could have done. Yoga, I’ve tried a couple times in the last six months… terrible idea. All the positions. I get really dizzy.	2:3 (High)
*Theme 2: Variance in understanding of the role of exercise in managing POTS*	
I think all of that [exercise] could over time help train your body to, well I don’t know if it actually would help train the body because they’re chronic conditions… So I don’t really know what the correlation is but I know that those are some of the benefits of exercise in general…	1:2 (Low)
My understanding is that we can approach treating ourselves with exercise from several directions at the same time. One would be venous return… The other part of it is increasing our blood volume… if we could work out like an endurance athlete, we could get the same adaptations they get.	2:1 (High)
I think from what I’ve heard, deconditioning can cause a temporary POTS-type condition… But for people who think all POTS is just deconditioning doesn’t make sense to me. I had a full six-pack when I got POTS. I was working out constantly.	3:1 (Low)
*Theme 3: Influence of experience on motivation*	
But he [cardiologist] talked to me about exercise pretty early on, but I was already doing a lot of it… I knew it inherently. I knew that I felt better when I ran. I had no idea why… I would be really diligent about it… because I just knew I would feel better if I did it.	8:3 (High)
Even in the best situation… medicated, hydrated, the whole thing, three minutes at the slowest pace I could possibly do, I tolerated it in the moment and then hours later I would feel sick and I would be sick for several days. I basically couldn’t get it slow enough… The activity I would be doing over the next several days that I’m cutting out just to do one minute, it just didn’t make sense.	7:3 (Low)
I tried to exercise my way out of it when I first got sick because I’m a PT and I thought exercise cured everything… And I did that [daily walking] for three years and it just never improved.	3:2 (Low)
*Theme 4: Barriers*	
[S]ome days I just can’t get out of bed… being able to come to terms with that and then return back to exercise when that period is over, I would say that is the hardest part. But objectively, and as someone who’s trained as a scientist, I see the value of exercise, I feel it. But then being able to put that into action when you’re not feeling well, it’s a much harder barrier to climb.	6:2 (High)
For me, a lot of it is my EDS. It’s a lot of joint pain and heart rate. So running is a no, both heart and joint pain wise it’s just so bad… there’s a lot of exercises that I can’t do just depending on the angle of your joints because I don’t want to pull them or twist.	1:4 (High)
[E]xercise is so important, but it can be extremely difficult to achieve when you work full time, especially when you have a demanding job. Unfortunately people have to choose between their livelihood and really living.	Survey
*Theme 5: Importance of social support*	
My husband’s very supportive so that it really helps… He’ll go on these walks with me so that if I go too far and I can’t walk back, let me ride his back to the car.	4:1 (High)
[I] have a POTS and ME/CFS aware exercise physiologist who has helped me to use Fitbit data (steps, HRV, heart rate) to manage my energy expenditure within my envelope. [B]ecause of his help I’ve been able to increase from 40 min of activity per day to 70 min per day which is a significant change in quality of life.	Survey
I think if I had to choose one thing that I think could really help me be able to be more consistent about the exercise, it would be hands down having a personal trainer or physical therapist or someone who is informed about POTS or in dysautonomia in general that can be there to monitor symptoms and know and understand when to ease up and when to push us harder.	1:2 (Low)
*Theme 6: Medical treatment needs for facilitating exercise*	
[W]hen I finally got medications… earlier this year, I was finally able to actually exercise, meaning I can actually sweat and do more rigorous exercise. So now I do exercise regularly more than I ever have in my entire life.	6:3 (High)
I had better experiences when I was doing physical therapy with EDS knowledgeable providers so they could manage any form issues and I was able to tap out and be assured I was still doing okay or meeting goals… We [people with EDS] fall through the cracks and when I try to exercise alone I always end up injured or overworked and end up worse than where I began.	Survey
I had an amazing cardiologist that referred me to the Levine program. Because of MCAS, I got much more symptomatic with exercise. After MCAS was under control, I was able to exercise regularly beyond what the Levine protocol recommended.	Survey
*Theme 7: Access needs*	
I think with some of those barriers it makes it hard to also do something consistently. But I know for myself in an ideal situation I would probably swim a couple times per week in a lap pool because one, I find that that helps myself with the thermal regulation situation.	1:2 (Low)
I’m very blessed that I was able to not have to work and that we are able to get the exercise equipment I needed because I can see barriers to being able to exercise. I couldn’t get to the gym. I couldn’t drive, I couldn’t walk.	2:3 (High)
*Theme 8: Exercise recommendations and protocols*	
I was recommended exercise for years and went to physical therapy for recumbent exercise. I bought a recumbent bike. This was all for POTS. [B]ut I had trouble implementing without the structure of CHOP, which I discovered around 2018.	Survey
The way that these structured programs are written is pretty perfectionistic. It’s like you need to do everything this week, and if you don’t do it this week, you have to repeat the week. And that sounds like punishment, the way that’s phrased. That’s not a positive reward.	6:1 (High)
Gently explain to patients that you know they feel like death but exercise could help. It’s just hard to hear over and over and over that I should exercise when I don't have the energy to exercise, shower take care of my kids and so on.	Survey
*Theme 9: Need for consistency and planning*	
The trick with that though is like you stop for three days and it’s over. That effect is bleeding and if you don’t maintain the schedule, it goes away very quickly and you kind of have to start back at zero if you take a week off for being sick or whatever.	2:1 (High)
Now even if I’m having a not so great POTS day or whatever, if I’m extra fatigued, if I even just do light exercise, not even trying to get to certain cardio levels, but I do it for 20 or 30 min, I feel better the next day. So that’s why I feel dependent on it.	8:4 (High)
*Theme 10: Role of pacing and flexibility*	
Yeah, you definitely have to plan. And you have to flexible with your plans, you maybe plan, like today, I woke up and I planned to do my Peloton bike ride, and do a strong workout, and I woke up, and didn’t feel like I could do that. So it’s to change the plan, and be flexible with your planning, and select from the menu of options that you know is safe.	4:1 (High)
I think for me it’s just balancing your energy out during the day. That’s the most important thing I’ve learned… if I do exercising, I have to be laying down for a minute, or a half an hour, to get rest and then I can continue… You have 50% of your energy as normal people do.	4:2 (High)
*Theme 11: Self efficacy*	
To go from never exercising to needing to exercise regularly just doesn’t seem feasible for me. I’m not sure my doctor understands how distressing that ask is for me.	Survey
I organize my life around it, I think because it’s probably my highest area of agency over this thing. At the beginning it was the only thing… exercise felt like a way to start getting better… I can mark it, I can see progress.	8:4 (High)

*EDS* Ehlers danlos syndrome,* HRV* heart rate variability, *MCAS* mast cell activation syndrome, *ME/CFS* myalgic encephalomyelitis, * POTS * postural orthostatic tachycardia syndrome, *CHOP* Children's Hospital of Philadelphia [POTS exercise protocol]

#### Personal factors: experience, knowledge, and barriers

##### Theme 1: Diversity in exercise experience, engagement, and preferences

The diversity in premorbid and current exercise frequency that emerged in the survey results was echoed in focus groups, with many participants across groups sharing that they had always been very active and many describing themselves as former, or lifelong, athletes. Conversely, others shared that they had never regularly exercised. Some shared that they simply have never enjoyed exercise, while others shared that exercise had always been difficult or caused them to feel bad physically.

Similarly, there was clear diversity in what kinds of physical activity focus group participants reported liking and tolerating. For example, some participants shared that slow walking is one of the most difficult activities for them, while others shared that slow, gentle walking is most tolerable. Similarly, some participants shared that they find strength training most beneficial, while others stated they cannot tolerate it. Additionally, several participants shared that they cannot tolerate yoga or tai chi, while others reported liking yoga and finding it helpful.

##### Theme 2: Variance in understanding of the role of exercise in POTS

A wide variation of knowledge about the role of exercise in managing POTS was described in the focus groups, with some expressing having no or limited understanding. Others expressed having some understanding that exercise helps “retrain” the body or autonomic nervous system but not knowing specifics. Other participants, mostly in the high exercise groups, expressed detailed understanding of the physiological rationale for exercise in POTS, including expansion of blood volume, improvement of venous return, and increased cardiac output.

Participants also expressed frustration with the explanation of POTS being caused by deconditioning and the assumption of deconditioning as a rationale for exercise. Many participants shared that they exercised regularly and were very fit when their symptoms began, with some sharing that they never stopped exercising and yet were still symptomatic. Relatedly, participants expressed frustration with exercise being presented as a “cure” for POTS rather than an intensive, ongoing treatment.

##### Theme 3: Influence of experience on motivation

Many in the high exercise groups reported that a primary motivator for exercise is the experience that it is helpful. Some shared figuring out on their own that their symptoms were under better control when they exercised regularly, while others shared that through the experience of completing a recommended exercise protocol, they saw their symptoms noticeably decrease.

Conversely, many inactive participants reported that exercise did not improve their symptoms or made them worse, despite having tried exercising consistently. Many of these participants identified themselves as having comorbid myalgic encephalomyelitis/chronic fatigue syndrome (ME/CFS), and some urged caution in prescribing exercise to those with POTS who may have ME/CFS.

##### Theme 4: Barriers: symptoms and time

Much of the discussion around barriers in both high and low exercise groups involved the impact of symptoms on exercise capacity. Many participants in the low exercise groups expressed believing in the value of exercise but struggling to engage due to their symptom burden and the dynamic nature of POTS. Participants discussed having days when they can not get out of bed, or have fainting episodes, gastrointestinal symptoms, or other severe symptoms. Others shared that they simply cannot engage in exercise at all, no matter how much they wish they could, due to overwhelming fatigue, post-exertional malaise, and/or exercise-provoked cardiopulmonary symptoms. Additionally, participants shared how comorbid symptoms, most frequently chronic pain, are additional barriers to exercise.

Others shared that time and life responsibilities, including work, school, and/or family responsibilities, make exercising challenging, with some specifying that they must reserve their limited energy and physical capacity for these tasks. Additionally, participants shared that the cumulative burden of chronic illness management makes it difficult to prioritize or fit in exercise.

#### Environmental factors: social support, medical intervention, access, and exercise recommendations and protocols

##### Theme 5: The importance of social support

The importance of support and guidance emerged as a theme across high and low exercise groups. Participants in high exercise groups shared how having supportive others in their personal lives (e.g., a partner to go on walks with) helps them exercise. Others shared how helpful it is to have support from knowledgeable healthcare professionals, including physical therapists or athletic trainers. When discussing what would help them exercise more, many in the low exercise groups expressed needing guidance from knowledgeable professionals, while others reported desiring others to exercise with.

##### Theme 6: Medical treatment needs for facilitating exercise

Another theme that emerged regarding facilitating factors was the role of medical intervention. Specifically, participants identified medication management and identification and treatment of comorbidities as critical needs that can support the ability to exercise. Several participants shared that starting medications was what made it possible for them to tolerate exercise. Participants also shared frustration that treatment guidelines can be interpreted as suggesting that medications should not be tried until and unless a patient has tried exercise. A less frequently mentioned related suggestion was intravenous hydration therapy immediately before exercise sessions.

As explained above, symptoms related to comorbid conditions were a commonly cited barrier. Some participants discussed not being able to exercise consistently because of the burden of their comorbidities (e.g., joint pain from Ehlers–Danlos syndrome), while others discussed how having comorbidities diagnosed and/or treated is what allowed them to begin or sustain regular exercise.

##### Theme 7: Access needs

While access to equipment and facilities was not one of the most frequently mentioned barriers in the survey, it emerged as a theme in qualitative analysis. Participants cited the difficulty of not having access to recumbent equipment and pools for swimming or other aquatic exercise. This theme often included mention of heat intolerance as a reason for needing indoor or water-based exercise options.

##### Theme 8: Exercise recommendations and protocols: helpful but can be improved

Across both low and high exercise groups, participants expressed believing that some kind of exercise protocol is necessary for guidance. Some participants shared trying to “figure out” exercise on their own and struggling to know what and how much to do. Others expressed finding the guidance available in protocols very helpful.

However, another theme that emerged across groups is that the protocols can be discouraging. Participants shared criticisms of protocols for requiring one to repeat or go backwards if they miss days. Some expressed that protocols begin at too challenging a level for those who do not regularly exercise, while others expressed that they may be too basic for those who are not deconditioned. Others pointed out that the amount of exercise recommended in the protocols adds up to more than is realistic for most people, i.e., hours per day. Particularly in the high exercise groups, participants shared suggestions for improvement of exercise guidance and protocols, including using technology, such as apps, building in more flexibility, and pairing protocols with individualized guidance.

Furthermore, participants expressed frustration with exercise recommendations that are given without further guidance or acknowledgement of barriers, including the severity of POTS symptom burden. Participants expressed wanting exercise recommendations to be delivered with compassion and understanding.

#### Behavioral factors: consistency, pacing, flexibility, and self-efficacy

##### Theme 9: Need for consistency and planning

A theme that emerged primarily in the high exercise groups and surveys was the importance of consistency and planning. Participants discussed experiencing that exercise helps them most when they engage in it consistently. Others emphasized the importance of proactive planning to prioritize regular exercise, including planning to conserve enough energy to be able to exercise. Furthermore, participants shared behavioral strategies that had been helpful for them be more consistent, including paying for an expensive gym membership, investing in fashionable exercise clothes, and choosing or planning exercise in a way that makes it easier to initiate.

##### Theme 10: Role of pacing and flexibility

Participants emphasized the importance of balancing consistency and planning with flexibility and pacing due to the dynamic nature of POTS. Some discussed needing to adjust exercise plans based on daily symptom burden, while others discussed needing to start or proceed far more slowly than they would have expected or that protocols specify.

##### Theme 11: Self-efficacy

Several sub-themes emerged relating to the relationship between self-efficacy and exercise. Some inactive participants voiced that they simply do not feel capable of exercising, due to symptoms, fatigue, or post-exertional malaise. Others shared that their self-efficacy for regular exercise has been negatively impacted by experiencing setbacks due to flares or injuries.

Conversely, other participants shared that one of their greatest motivators for continued engagement in exercise is that it gives them an increased sense of agency or control over their condition. Similarly, others shared experiences of finding their self-efficacy increase over time as they have made progress with exercise.

## Discussion

This study sought to elucidate the issue of exercise as a treatment for POTS through collecting patient perspectives via focus groups and an online survey. Findings show that adults with POTS are diverse in terms of their premorbid exercise engagement, with some exercising frequently and others rarely. We found that while current exercise levels were overall lower, the majority of participants reported some physical activity, with a small proportion reporting regular, high intensity exercise. Findings also identify factors that may influence how much someone with POTS exercises, including knowledge and beliefs about the role of exercise in managing POTS, personal and environmental barriers, personal and environmental facilitating factors, and behavioral self-regulation strategies. Across these domains a key finding emerged: most adults with POTS believe exercise is important in managing their condition and yet many struggle to do it at recommended levels. Participants reported significant barriers, including most saliently, their symptom burden, and needs, including guidance, support, medical treatment optimization, and behavioral skills.

These findings add important nuance and context to our understanding of the role of exercise in treating POTS. They provide an important first step in addressing the concerns about the existing body of research on exercise for POTS detailed in a recent systematic review [15] which concluded that while available evidence clearly supports a role for exercise in treating POTS, further research is needed that better reflects rates of comorbidity in the POTS population, involves individualized exercise programs, and includes symptom burden and functioning and not just cardiac parameters as primary outcomes.

Patient perspectives can inform guidelines both for how clinicians communicate about exercise and sequence it with other treatment recommendations and for the design of interventions to support individuals in engaging in exercise. Regarding communication about exercise recommendations, it is vital that clinicians not make assumptions about individuals’ past or current exercise engagement and to ask patients about their exercise experience, preferences, and specific barriers. Our findings suggest that clinicians should provide detailed physiological rationale for the specific benefits of exercise in POTS beyond countering deconditioning, framing it as an ongoing treatment and not a cure. Furthermore, our findings highlight the importance for clinicians who treat POTS to consider the impact of comorbid conditions and, as appropriate, refer the patient for diagnosis and treatment of suspected comorbidities. Additionally, clinicians should consider the potential for medication management to improve exercise tolerance among patients who report being unable to begin or sustain exercise training due to intolerance or symptoms. It is worth noting that research on exercise behavior has consistently shown that affective judgment constructs, including enjoyment and intrinsic motivation, are more effective motivators of exercise than expected health-related benefits [30], and thus it is crucial not to minimize the importance of individuals’ subjective experience during and immediately following exercise as motivating or deterring factors. Perhaps most importantly, participants expressed wanting clinicians to acknowledge just how difficult exercise is, especially in the context of high symptom burden. It is important to contextualize treatment recommendations in light of the overall challenges patients with POTS experience that are related to diagnostic delays, disbelief and dismissal, and the “invisible” nature of their condition [23].

It is likely not realistic to address many of these suggested needs in standard medical care. Rather, these point to a need for interventions that include multidisciplinary treatment including social and motivational support, paired with medical intervention aimed at reducing symptoms and increasing exercise tolerance while individuals build strength and stamina. Notably, several recent publications present, to our knowledge, the first published examples of more individualized exercise programs for POTS patients. Trimble et al. [9] describe a virtually delivered physical therapy intervention at their dysautonomia clinic, which is individualized, with significant education components, and emphasis on pacing, strengthening, and stabilization. Svensson et al. [31] present a feasibility study of an individualized application of existing POTS protocols for post-COVID POTS that included weekly sessions with a physiotherapist and adaptations to exercises, reporting a completion rate of 100% among their small sample, and improvements in physiological parameters and self-reported autonomic symptoms. Wheatley-Guy et al. [32] present a small randomized controlled trial comparing individualized exercise guidance to standard of care, reporting that exercise engagement was higher and improvements in physiological and symptom measures were superior in the guidance group.

Our study has several limitations. Because we used a convenience sample, there may be bias in who responded to recruitment materials, completed measures, and/or participated in focus groups, such as individuals with stronger negative or positive attitudes towards exercise being more motivated to participate. Moreover, while our total sample is relatively large and has some ethnic diversity, it may not be representative of the population of individuals with POTS in terms of factors such as age, comorbidities, or symptom burden. Also, it does not include the degree of ethnic and gender diversity needed to address the lack of representation in POTS research samples. Furthermore, our inclusion criteria of including confirmed diagnosis and all behavioral data, including exercise, is self-reported, which prior research has shown most individuals are not able to report accurately [33, 34]. Future studies could address this limitation by collecting data on enrolled participants using activity trackers. We also recognize flaws in our study design that emerged with analysis, including asking participants about their confidence in engaging in regular exercise using an amount that is consistent with general exercise recommendations (30 min for 3 times per week) but considerably lower than the amount prescribed in most POTS exercise protocols. It is possible that confidence for this greater amount of exercise could be lower. Additionally, we designed our survey before completing the focus groups and incorrectly assumed that participants would have only attempted exercise protocols if they had been prescribed them, and so did not ask follow-up questions to those who indicated they had never had a protocol prescribed or recommended. We learned in the focus groups and the qualitative survey data that many individuals reported finding protocols themselves. It is possible that completion rates would be higher among those who took the initiative to seek out exercise advice themselves.

## Conclusions and future directions

This study highlights the critical need to inquire about and listen to patient perspectives regarding lifestyle management of chronic illness, as a treatment is only effective if it is feasible and targets what matters to patients. The recent studies summarized in the above text suggest that individualized exercise guidance is feasible among patients with POTS and may result in higher rates of engagement. The next steps are to conduct larger trials evaluating individualized programs, potentially including motivational and support components. Further research is also needed to further elucidate which subsets of patients benefit from which kinds of exercise, at which doses, and in which ways. It is our hope that the voices of those most affected by this condition—the patients themselves—are included in the design and evaluation of treatment guidelines and interventions.

## Data Availability

The data that support the findings of this study are available from the corresponding author (EGW), upon reasonable request.
